# Artificial intelligence in nutritional oncology: From isolated screening tools to agentic intervention systems

**DOI:** 10.18632/oncotarget.28874

**Published:** 2026-05-04

**Authors:** Arnab Sarkar, Yashbir Singh-Wolkenhauer

**Keywords:** artificial intelligence, cancer

*Cancer-related malnutrition affects up to 80% of patients and contributes to 10 to 20% of cancer deaths, yet the registered dietitian-to-patient ratio in oncology stands at 1:2,308. AI tools for malnutrition screening, body composition analysis, and dietary counseling show promise in isolation but cannot reason across data modalities or adapt to evolving treatment courses. Agentic AI, a class of autonomous systems capable of reasoning, tool use, planning, and memory, has demonstrated near-clinical-grade performance in oncology decision support. We propose a multi-agent architecture for nutritional oncology governed by a graduated autonomy model, and discuss the evidentiary, regulatory, and equity barriers that must be addressed before clinical deployment.*

## The silent crisis in cancer nutrition

Malnutrition remains one of the most pervasive yet underaddressed complications in oncology [1]. It affects 40 to 80% of cancer patients depending on tumor type and stage, and is estimated to contribute to 10 to 20% of cancer deaths [2, 3]. The European Society for Clinical Nutrition and Metabolism (ESPEN) has documented the downstream cascade, including diminished treatment tolerance, prolonged hospitalizations, increased healthcare costs, and reduced survival [2, 3]. Cancer cachexia develops in up to 80% of patients with advanced disease and remains an independent predictor of mortality regardless of body mass index [3].

Despite decades of clinical nutrition guidelines, including ESPEN’s cancer nutrition guideline and ASPEN’s 2024 systematic review recommending validated malnutrition screening in all oncology outpatients [4], the implementation gap persists. A 2024 survey of oncology clinicians found that only 26% reported nutrition specialist integration into their multidisciplinary teams [5]. The registered dietitian-to-oncology-patient ratio in U.S. outpatient settings stands at approximately 1:2,308, a nineteen-fold shortfall from the estimated 1:120 needed for proactive nutritional care [6]. The consequence is predictable: only 12% of cancer survivors met recommended fruit and vegetable intake criteria [7]. This is not merely a knowledge deficit; it is a systems failure, and precisely the kind that artificial intelligence is now poised to address.

## The current landscape: Pattern recognition without action

The application of AI to nutritional oncology has advanced considerably over the past five years, though largely within the confines of pattern recognition. Machine learning models, including random forests, support vector machines, and gradient-boosted classifiers, now detect malnutrition risk with areas under the curve (AUC) exceeding 0.80, achieving performance comparable to or exceeding conventional screening instruments such as the NRS-2002 and PG-SGA [2]. Deep learning algorithms applied to routine computed tomography scans enable automated segmentation of skeletal muscle and adipose tissue at the L3 vertebral level with high accuracy, supporting opportunistic detection of sarcopenia from imaging already obtained for staging [2, 8].

On the patient-facing side, Ina, an AI-based virtual dietitian deployed nationally across all 50 U.S. states, reported that 84% of 3,310 cancer patients used the advice to guide their diet, with 88% reporting improved symptom management and 94% expressing satisfaction over a median retention of 8.8 months [9, 10]. A 2025 ASCO presentation subsequently established the equivalence of Ina’s guidance to that of human registered dietitians [11]. These metrics, however, derive from an observational deployment of a commercial product rather than a randomized trial, and should be interpreted with caution given potential selection and attrition bias.

These achievements are meaningful, but they share a fundamental limitation: each tool operates in isolation, performing a single task (screening, segmenting, or advising) without the capacity to reason across data modalities, coordinate interventions, or adapt to the evolving clinical trajectory of a patient undergoing active treatment. They identify risk. They do not close the loop.

## The agentic paradigm: From passive models to autonomous reasoning

A new class of AI systems, termed agentic AI, offers a fundamentally different architecture. Unlike traditional machine learning models that classify inputs into outputs, or generative AI that responds to individual prompts, agentic AI systems reason autonomously over complex problems, invoke external tools, maintain memory across interactions, and adapt their strategies based on observed outcomes [12, 13].

The distinction is functional, not merely technical. Where a conventional model answers the question “Is this patient malnourished?”, an agentic system pursues the goal “Optimize this patient’s nutritional status throughout their treatment course,” autonomously decomposing that objective into sensing, reasoning, and acting steps.

The theoretical framework rests on four design patterns formalized in the AI literature [12]: (1) reasoning, generating chains of clinical logic before acting (for example, “Weight down 4 kg in two weeks, prealbumin declining, FOLFOX with mucositis risk: assess oral intake and adjust caloric supplementation”); (2) tool use, dynamically querying electronic health records, laboratory systems, and clinical guidelines; (3) planning, decomposing clinical objectives into executable subtasks across days to months; and (4) memory, maintaining longitudinal awareness of a patient’s dietary preferences, treatment responses, and nutritional trajectory.

Early evidence suggests agentic AI can deliver meaningful clinical performance in oncology. Ferber et al. demonstrated in *Nature Cancer* that an autonomous AI agent integrating GPT-4 with eight precision oncology tools achieved 91.0% clinical accuracy and 87.2% completeness on multimodal gastrointestinal cancer cases, versus 30.3% for the language model alone [14]. Oxford’s TrustedMDT, a three-agent system for multidisciplinary tumor boards, entered clinical piloting in early 2026 [15]. At ASCO 2025, a multi-agent framework using three coordinated AI agents achieved 93% accuracy in guideline selection and 88% in clinical question answering, outperforming standalone foundation models by a factor of two [16]. A comprehensive review in *Nature Reviews Cancer* has since mapped the broader landscape [13].

These results demonstrate near-clinical-grade performance under constrained conditions. What remains is the application to nutritional oncology, and a framework for governing how much autonomy these systems should exercise. We propose a graduated autonomy model suited to the risk profile of nutritional decisions: low-risk actions (recipe recommendations, symptom-triggered dietary tips) may operate with minimal oversight; moderate-risk actions (caloric target adjustments, dietitian referral triggers) require confidence thresholds and human review; and high-risk actions (initiation of enteral or parenteral nutrition) demand explicit clinician authorization.

## Envisioning the agentic nutritional oncology system

Nutritional oncology is uniquely suited for agentic AI integration, demanding continuous monitoring, coordination among care team members, real-time adaptation to treatment side effects, and personalization across dietary, cultural, and genomic dimensions.

**Figure 1:**
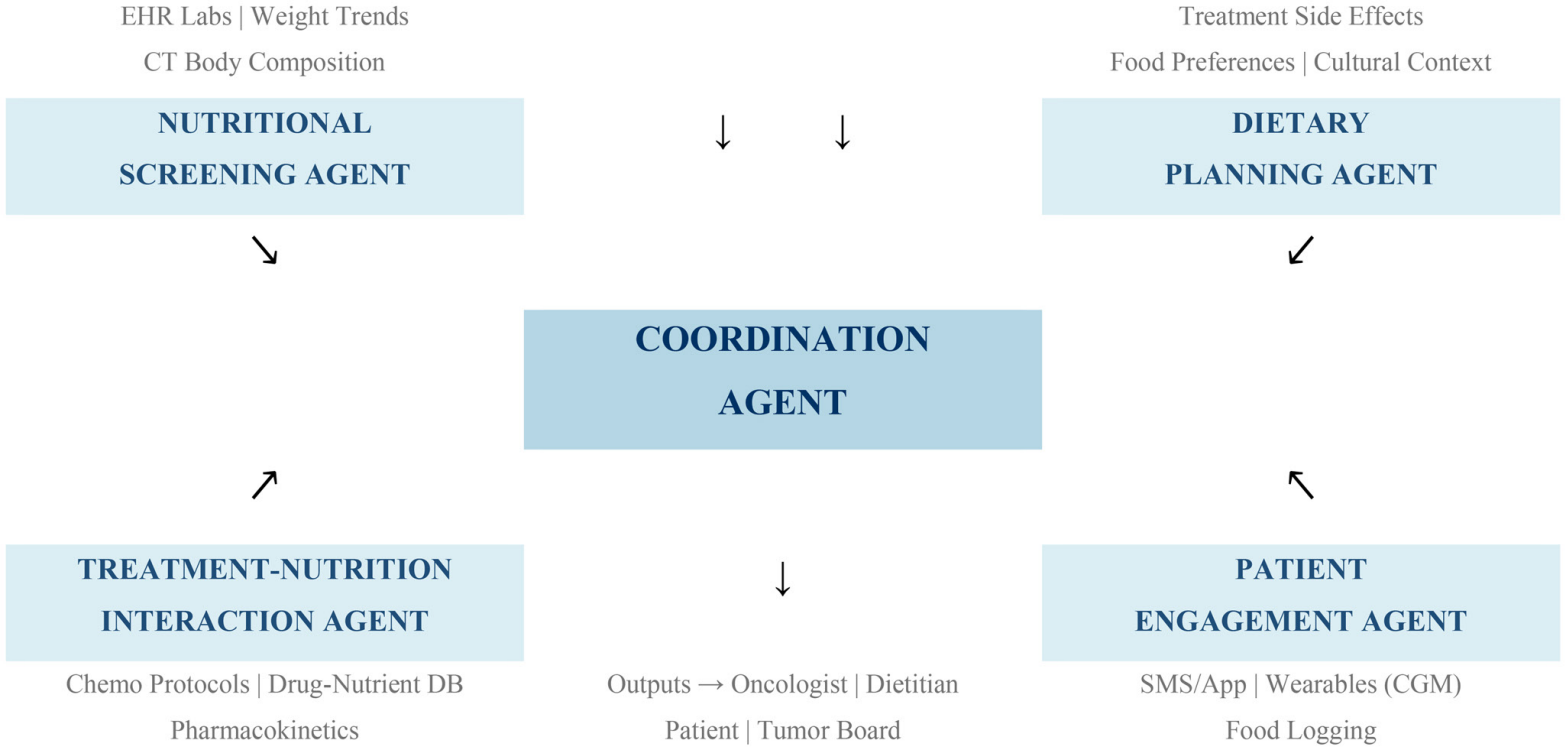
Proposed multi-agent architecture for agentic nutritional oncology. The Coordination Agent orchestrates four specialized agents, each drawing on distinct data sources, with graduated human oversight governing the autonomy of clinical actions.

A multi-agent architecture could be structured as follows (Figure 1).

A *Nutritional Screening Agent* would integrate laboratory values (prealbumin, C-reactive protein, weight trajectories) and CT-derived body composition metrics to detect early cachexia signals before clinical malnutrition becomes apparent. A *Dietary Planning Agent* would generate personalized meal plans responsive to treatment side effects (mucositis, dysgeusia, nausea), food preferences, and cultural context, adjusting daily rather than at quarterly clinic visits. A *Treatment-Nutrition Interaction Agent* would monitor drug-nutrient interactions, optimize timing of nutritional interventions relative to chemotherapy cycles, and flag contraindicated supplements. A *Patient Engagement Agent*, operating through conversational interfaces similar to Ina’s SMS model, would deliver dietary coaching, track adherence via food logging, and integrate wearable data. When conflicts arise between agents (for instance, a caloric supplementation that conflicts with a drug-timing constraint), a *Coordination Agent* would apply predefined resolution rules and orchestrate the overall system, escalating to clinician review when thresholds are crossed.

In practice, such a system would embed within existing clinical infrastructure: nutrition summaries would appear in the EHR inbox, body composition trends would populate tumor board packets, and patients would receive SMS-based guidance between clinic visits.

The Food4Me trial demonstrated across seven European countries that personalized nutrition advice significantly outperformed generic dietary guidance [17], and the PREDICT study established that individual postprandial responses to identical foods vary enormously even among genetically similar individuals [18]. Multi-omics AI models are achieving robust predictive capability (pooled AUC 0.81 across 42 studies) for cancer-specific metabolic signatures by integrating genomic, transcriptomic, proteomic, and dietary exposure data [9]. Embedded within an agentic framework, these models could generate individualized nutritional risk profiles that dynamically inform intervention, connecting dietary intake to validated clinical endpoints through continuous feedback loops.

## Challenges and the path forward

Significant barriers must be acknowledged. Even the highest-performing agentic system produced correct responses in 91% of cases (223 of 245), with 6.5% classified as incorrect but not harmful and an additional 2.4% deemed potentially harmful [14]. Hallucination rates in large language models range from 15 to 41% across studies, demanding robust guardrails [19]. The risk of automation complacency, where clinicians defer uncritically to algorithmic recommendations, must also be carefully managed. No autonomous AI agent has received regulatory clearance from the U.S. Food and Drug Administration for independent clinical decision-making [20]. The European Union AI Act conditionally classifies medical AI as high-risk, with a phased compliance timeline extending to 2027, mandating human oversight for autonomous clinical decisions [21]. Zero randomized controlled trials have evaluated AI-driven nutritional interventions against hard oncological endpoints such as survival, treatment completion, or dose intensity maintenance [2]. This evidentiary gap must be addressed before agentic nutritional AI can transition from concept to clinical standard.

The aggregated data surface required by such systems (EHR records, imaging, wearable streams, and patient-reported dietary data) raises significant privacy considerations under HIPAA, and the graduated autonomy model introduces unresolved questions about clinical liability when errors occur at different levels of autonomous action.

Equally important are health equity considerations. Dietary patterns are deeply embedded in cultural, religious, and socioeconomic contexts. AI systems trained predominantly on Western dietary data risk perpetuating algorithmic bias [19]. Any agentic nutritional system must be validated across diverse populations and designed for equitable access, particularly in low-resource settings where the workforce gap is most acute.

## CONCLUSIONS

The technical foundations for agentic AI in oncology are in place. Nutritional oncology, with its workforce crisis, fragmented care pathways, proven AI feasibility, and the devastating yet preventable toll of cancer-related malnutrition, represents a compelling next frontier. We call for nutrition-specific agent modules within existing oncology AI orchestrators, prospective validation through randomized trials with patient-centered endpoints, and integration of nutritional AI agents into multidisciplinary tumor board workflows. The building blocks exist; what is now needed is rigorous validation and the collaborative will to assemble them.
